# Why do ‘OFF’ periods still occur during continuous drug delivery in Parkinson’s disease?

**DOI:** 10.1186/s40035-022-00317-x

**Published:** 2022-10-13

**Authors:** Silvia Rota, Daniele Urso, Daniel J. van Wamelen, Valentina Leta, Iro Boura, Per Odin, Alberto J. Espay, Peter Jenner, K. Ray Chaudhuri

**Affiliations:** 1 Department of Basic and Clinical Neurosciences, Institute of Psychiatry, Psychology and Neuroscience, King’s College London, London, UK; 2 Parkinson’s Foundation Centre of Excellence, King’s College Hospital, London, UK; 3 Department of Neuroimaging, Institute of Psychiatry, Psychology and Neuroscience, King’s College London, London, UK; 4 Centre for Neurodegenerative Diseases and the Aging Brain, Department of Clinical Research in Neurology, University of Bari ‘Aldo Moro, “Pia Fondazione Cardinale G. Panico”, 73039 Tricase, Italy; 5 Donders Institute for Brain, Cognition and Behaviour, Department of Neurology, Radboud University Medical Centre, Nijmegen, The Netherlands; 6 School of Medicine, University of Crete, Crete, Greece; 7 Department of Neurology, University Hospital of Heraklion, Crete, Greece; 8 Division of Neurology, Department of Clinical Sciences Lund, Lund University, Lund, Sweden; 9 University of Cincinnati Gardner Neuroscience Institute, Gardner Family Center for Parkinson’s Disease and Movement Disorders, Department of Neurology, University of Cincinnati, Cincinnati, OH USA; 10 Institute of Pharmaceutical Sciences, Faculty of Life Science and Medicine, King’s College London, London, UK

**Keywords:** ‘OFF’ periods, Continuous drug delivery, Continuous dopaminergic stimulation, Rotigotine patch, Subcutaneous apomorphine infusion, Levodopa-carbidopa intestinal gel

## Abstract

Continuous drug delivery (CDD) is used in moderately advanced and late-stage Parkinson’s disease (PD) to control motor and non-motor fluctuations (‘OFF’ periods). Transdermal rotigotine is indicated for early fluctuations, while subcutaneous apomorphine infusion and levodopa-carbidopa intestinal gel are utilised in advanced PD. All three strategies are considered examples of continuous dopaminergic stimulation achieved through CDD. A central premise of the CDD is to achieve stable control of the parkinsonian motor and non-motor states and avoid emergence of ‘OFF’ periods. However, data suggest that despite their efficacy in reducing the number and duration of ‘OFF’ periods, these strategies still do not prevent ‘OFF’ periods in the middle to late stages of PD, thus contradicting the widely held concepts of continuous drug delivery and continuous dopaminergic stimulation. Why these emergent ‘OFF’ periods still occur is unknown. In this review, we analyse the potential reasons for their persistence. The contribution of drug- and device-related involvement, and the problems related to site-specific drug delivery are analysed. We propose that changes in dopaminergic and non-dopaminergic mechanisms in the basal ganglia might render these persistent ‘OFF’ periods unresponsive to dopaminergic therapy delivered via CDD.

## Introduction

**Fig. 1:**
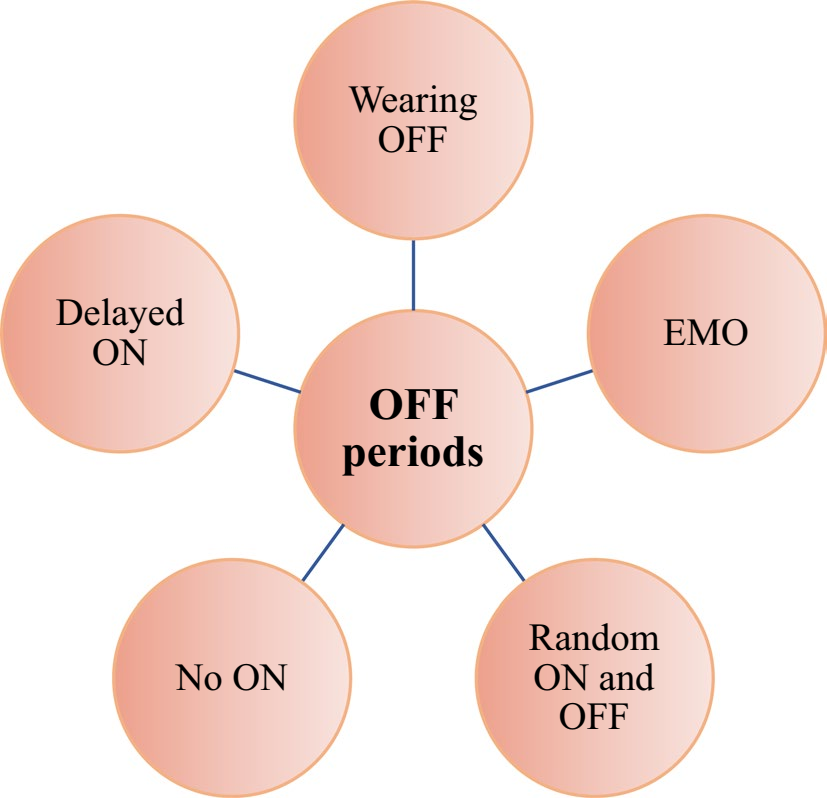
OFF periods in Parkinson’s disease. EMO: early morning off

Response fluctuations (motor and non-motor ‘OFF’ periods) are a common feature of levodopa-treated Parkinson’s disease (PD) and major determinants of quality of life in PD, serving as a main outcome measure in most key clinical trials [1]. They occur in both early and late illness, with reported onset as early as 5–6 months following the initiation of levodopa treatment [2–4] and are characterised by ‘OFF’ periods that are clinically heterogeneous. They manifest as ‘wearing OFF’, early morning ‘OFF’, delayed ON, no ON or random/unpredictable ‘ON–OFF’ [5] (Fig. 1). The duration of ‘OFF’ periods, as well as their severity and unpredictability are the features most associated with a more impaired quality of life [6–8]. Predictable ‘OFF’ periods can be associated with drug dose and the onset of ‘wearing OFF’ with the end of its effect [9]. Loss of drug efficacy characterising predictable ‘OFF’ periods has been associated with a loss of presynaptic storage of levodopa/dopamine in remaining dopaminergic terminals in the striatum with increased disease severity [5, 10–12]. This is, however, unlikely to be the entire explanation, as ‘wearing OFF’ is also reported with dopamine agonists and in animal models of PD without presynaptic involvement [12–15]. A post-synaptic component affecting basal ganglia output seems likely and this may be associated with the loss of the long-duration response to levodopa, although this has recently been disputed [5, 12, 16–19]. Unpredictable or random ‘OFF’ periods are a more complex phenomenon and difficult to treat with dopaminergic medications, even when given continuously to achieve continuous drug delivery (CDD) [9]. Factors affecting the peripheral pharmacokinetic profile of levodopa, such as the interference of high-protein meals, gastroparesis, *H. Pylori* infection or constipation, can contribute to the unpredictability [20, 21]. However, the central mechanisms underlying unpredictable ‘OFF’ periods, such as ‘ON–OFF’, may involve non-dopaminergic pathways, although there has been no preclinical investigation examining whether or why these occur.

Standard therapies for treating ‘OFF’ periods involve alterations of the dose, dose frequency and timing of oral dopaminergic medications, and the use of adjuncts (enzyme inhibitors and agonists) to levodopa to extend its duration of effect [5, 22]. While these reduce ‘OFF’ periods and ‘OFF’ time in the short term, they are not effective in producing a constant restoration of motor or non-motor function. Dosing of levodopa using new slow-release or longer-acting preparations as well as dopamine agonists, has largely failed to provide continuous dopaminergic stimulation (CDS) or to restore ‘ON’ periods without troublesome dyskinesia [23]. Indeed, avoiding the standard oral administration of dopaminergic drugs has been seen as essential in achieving more predictable delivery of dopaminergic medications and more predictable ‘ON’ time. In this respect, the concept of CDS has proven useful [24, 25]. While initially proposed to provide a more physiological dopaminergic response that avoids the onset of motor fluctuations and motor complications, it has morphed in to providing a means of CDD that has proven effective in reducing ‘OFF’ time and lessening the intensity of existing dyskinesia [5], even if it fails to achieve a complete control of motor fluctuations.

Three current distinct approaches to non-oral CDD have been introduced: the transdermal delivery of rotigotine, predominantly used in early PD, the continuous subcutaneous infusion of apomorphine (CSAI), and the intraduodenal delivery of levodopa-carbidopa intestinal gel (LCIG), recently available in a new formulation with entacapone (levodopa–entacapone–carbidopa intestinal gel) [26], indicated for the treatment of advanced PD [25, 27]. Additionally, novel formulations of subcutaneously-delivered levodopa are currently under investigation [28]. The use of alternative routes to the oral one, is likely to be the key factor for improved outcomes, as they avoid the gastrointestinal route and thus overcome drug delivery bottlenecks [29].

In early PD, rotigotine transdermal patches are employed as a mono- or adjunctive therapy to improve motor function as well as motor and non-motor fluctuations, in particular ‘wearing OFF’ [30–32]. This strategy reduces but does not abolish ‘OFF’ periods [33]. In later PD, CSAI and LCIG are used, and controlled data from pivotal studies suggest a reduction in ‘OFF’ time and an increase in ‘ON’ time without troublesome dyskinesia in the short term [34–37]. However, after a variable “honeymoon” period, most patients still experience ‘OFF’ episodes despite receiving CDD [38]. Although the technologies involved, the device-related issues or the complications at the site of infusion may explain the emergent and “refractory” ‘OFF’ periods, they are unlikely the sole reason. The possibility of involvement of non-dopaminergic pathways needs to be explored.

### The problem of drug-resistant motor fluctuations

There remains a fundamental issue as to why drug-resistant fluctuations and ‘OFF’ periods occur despite CDD. As an example, multiple randomised, double-blind, placebo-controlled trials have reported improved motor and non-motor symptoms with transdermal rotigotine as an early monotherapy [30, 32, 39, 40] and as an adjunctive therapy in advanced PD [33, 41–45]. This strategy is based on preclinical studies where sustained delivery of rotigotine provided stable extracellular drug levels in the striatum, resulting in continuous stimulation of dopamine receptors [46]. When translated into PD patients, continuous transdermal drug delivery of rotigotine results in stable plasma levels over a 24-h period [47], but whether this translates into CDS in the basal ganglia remains unclear, even though preclinical data underpin this concept. While there is a sustained improvement in ‘OFF’ time and reduction in absolute ‘OFF’ time with rotigotine, motor fluctuations are not abolished and more than 50% of motor ‘OFF’ time remains across the patient population studied (Table 1). The effects of transdermal rotigotine on non-motor fluctuations, with the exception of pain [48], are unknown and need to be evaluated [49, 50], although it is presumed that the non-motor fluctuations are likely also to be prevalent during these emergent “refractory” ‘OFF’ periods, as non-motor fluctuations are often associated with motor fluctuations, particularly with the ‘OFF’ state [1, 51]. Specific reasons for a failure to eliminate ‘OFF’ periods could be application site reactions, which also represent the major reason for discontinuation [52], or reduced patch adhesion [53]. Additionally, there could potentially be differences in the extent of rotigotine delivery through the skin and to the systemic circulation and then to the brain between individual PD patients, but these have not been defined. It could be argued that the rotigotine dose employed in the patches is inadequate to provide plasma/brain levels of the drug to abolish ‘OFF’ periods, but these also persist in patient populations receiving concomitant oral dopaminergic therapy. Finally, it is reasonable to assume that the “refractory” ‘OFF’ periods during rotigotine treatment are qualitatively more severe than those present with apomorphine or levodopa. In fact, despite the lack of specific studies comparing ‘OFF’ quality between the different regimes, it is well known that levodopa treatment is associated with better motor function and quality of life when compared to dopamine agonists [54, 55], with the exception of apomorphine which has been confirmed to be as effective as levodopa [56, 57].

**Table 1:** Reduction of daily ‘OFF’ time with rotigotine patch treatment

Study	Number of participants	Average follow-up duration (months)	Reduction of daily ‘OFF’ time (%)
LeWitt et al. [41]	168	6	37
Poewe et al. [44]	204	6	25
Nicholas et al. [33]	397	4	34
Mizuno et al. [42]	110	5	31
Nomoto et al. [43]	54	3	32
Zhang et al. [45]	170	3	34
Weighted average improvement in OFF time*			32.4

Data from controlled studies assessing the efficacy of rotigotine transdermal patch in reducing ‘OFF’ time in the treatment of patients with advanced disease. *Weighted for participant number per study

CSAI is indicated for patients with unpredictable or prolonged ‘OFF’ time, motor fluctuations and dyskinesia [58]. The efficacy of CSAI, in monotherapy or as add-on to levodopa, in reducing the ‘OFF’ time has been demonstrated in several uncontrolled open-label series (Table 2). The average ‘OFF’ time reduction was 60%, while the average reduction in dyskinesia severity was 30% [59]. This was confirmed in the TOLEDO study, the first-ever randomised, parallel-group, double-blind, placebo-controlled, multi-centre trial examining CSAI over 12 weeks in advanced PD [36]. The study showed that apomorphine significantly reduced ‘OFF’ time compared to placebo (− 2.47 vs − 0.58 h/day), without increasing troublesome dyskinesias, and allowed a dose reduction of concomitant oral antiparkinsonian medications. These effects were maintained in the open-label extension of the TOLEDO study over 52 weeks [60]. The effects of CSAI were comparable to LCIG in terms of efficacy in treating motor fluctuations in advanced PD [61], but once again without the abolition of ‘OFF’ time, despite optimisation of both apomoprphine delivery and concommitant oral or transdermal therapy. Although apomorphine is also beneficial in treating some non-motor symptoms of PD, such as mood or cognition [62, 63], there is no evidence on the treatment of non-motor fluctuations occurring during the ‘OFF’ state. The persistence of ‘OFF’ periods despite the clear efficacy of CSAI in advanced PD, can be due, as with rotigotine, to the under-dosing, although ‘OFF’ periods are emergent even with high-dose infusion of apomorphine, either in monotherapy or as an add-on to oral levodopa, as in most of the studies which have evaluated apomorphine efficacy [64]. It is feasible that occasional technical issues related to the device, such as pump failure, line blockage or inaccurate needle insertion, can cause a reduction of CSAI efficacy [61, 65]. The extent of apomorphine delivery from the subcutaneous site to the systemic circulation may be determined by the site of injection (the abdomen seems to be the best site), the state of the skin (warm skin increases the absorption compared to cold skin), and volume and depth of the injection (a greater volume is associated with a better absorption) [61, 66]. But these are unlikely to explain the persistence of ‘OFF’ periods across the patient populations studied. Finally, the occurrence of subcutaneous nodules due to an inflammatory reaction [61, 67] can mechanically affect local absorption directly or by altering the blood flow [61]. But again, this seems unlikely a cause of the persistence of significant ‘OFF’ time.

**Table 2:** Reduction of daily ‘OFF’ time with continuous subcutaneous apomorphine infusion treatment

Study	Number of participants	Average follow-up duration (months)	Reduction of daily ‘OFF’ time (%)
Stibe et al. [68]	11	8	62
Chaudhuri et al. [69]	7	11	85
Frankel et al. [70]	25	22	55
Pollak et al. [71]	9	10	67
Hughes et al. [72] ±	22	36	59
Stocchi et al. [73]	10	12	58
Poewe et al. [74]	18	20	58
Kreczy-Kleedorfer et al. [75]	14	26	77
Gancher et al. [76]	6	3	58
Colzi et al. [77] ±	19	35	72
Pietz et al. [78] ±	25	44	50
Wenning et al. [79] ±	16	57	55
Kanovsky et al. [80]	12	24	80
Manson et al. [81] ±	64	34	49
Di Rosa et al. [82]	12	12	40
Morgante et al. [83]	12	24	60
Katzenschlager et al. [84]	12	6	38
De Gaspari et al. [85]	13	12	51
Garcia-Ruiz et al. [86]	82	20	80
Martinez-Martin et al. [87]	17	6	65
Antonini et al. [88]	12	60	49
Drapier et al. [89]	23	12	36
Borgemeester et al. [90]	45	26	45
Sesar et al. [91]	230	26	78
Sesar et al. [92]*	18	16	74
Papuc et al. [93] ±	9	24	86
Isaacson et al. [94]	99	3	47
Katzenschlager et al. [60]	84	52	53
Weighted average improvement in OFF time**			62.4

Data from open-label studies assessing the efficacy of continuous subcutaneous apomorphine infusion (CSAI) in reducing ‘OFF’ time in the treatment of patients with advanced Parkinson’s disease. Only studies with reported reduction of daily ‘OFF’ time were included

±Studies in which CSAI monotherapy was achieved in the whole cohort or in a sub-group of patients

*Only the cohort before deep brain stimulation has been included

**Weighted for participant number per study

In patients with advanced PD and severe motor fluctuations, continuous delivery of LCIG directly to its site of absorption in the duodenum avoids the typical fluctuations in plasma profile associated with conventional oral levodopa formulations, which lead to a non-physiological pulsatile stimulation of striatal dopamine receptors and are associated with levodopa-induced motor fluctuations and complications [24]. Pharmacokinetic studies have shown a significantly reduced intra-subject variability in plasma levels over the period of infusion compared to that during oral treatment [95, 96]. The continuous delivery of levodopa is expected to markedly reduce ‘OFF’ periods in advanced PD compared to oral therapy, and this has been confirmed in a systematic review and meta-analysis [97], and in an exhaustive qualitative analysis of 27 studies with mixed designs in which ‘OFF’ time outcome measures were reported ≥ 12 months after the initiation of LCIG [98] (Table 3). The ‘OFF’ time showed a mean relative reduction of 47%–82% at 3–6 months of follow-up and up to 83% at 3–5 years of follow-up [98]. However, as with other continuous delivery systems, ‘OFF’ time was not abolished and similarly, the effects on non-motor fluctuations have not been studied, despite the proven efficacy on specific non-motor symptoms such as sleep, fatigue, and gastrointestinal symptoms [62, 63]. Despite the reductions of ‘OFF’ time, the persistence of ‘OFF’ periods during LCIG therapy can be due to several factors, mostly overlapping with those described for CSAI. The LCIG device-related issues such as tube dislocation and pump malfunctioning, or peristomal complications such as the presence of granulation tissue, skin problems or local infection, might further contribute to ‘OFF’ persistence [99, 100]. In addition, comorbid *H. Pylori* infection as well as alterations of gut microbiota, including small intestinal bacteria overgrowth (SIBO) and high prevalence of tyrosine decarboxylase-producing bacteria, can interfere with LCIG absorption, ultimately leading to motor complications and persistence of ‘OFF’ periods [29, 101–103]. Constipation might contribute to LCIG erratic gut absorption [104], and protein-rich meals (containing large neutral amino acid) can worsen the parkinsonian symptoms, because of levodopa fluctuations in the brain [103, 105]. On the other side, fasting can also contribute to the worsening of motor fluctuations. A recent study has demonstrated that the maintenance dose of LCIG is strongly correlated with the mean plasma concentration of levodopa in the absence or presence of lunch, and comparison of the pharmacokinetic parameters showed that the coefficient of variation is significantly greater in fasting patients than in those that did eat [106]. Finally, it has been demonstrated that LCIG treatment is associated with high plasma levels of 3-O-methyldopa (3-OMD), a metabolite of levodopa (converted by catechol-O-methyltransferase [COMT]) which competes with levodopa itself for brain penetration, a phenomenon that can be counteracted by the concomitant administration of a COMT inhibitor [107, 108]. However, even when all these factors are taken into consideration for optimization of LCIG administration, the persistence of ‘OFF’ periods is still commonly observed in everyday clinical practice. It should be noted that subcutaneous levodopa infusion, which is currently under investigation, might be a valuable alternative option to LCIG, as it does not require intrajejunal tube insertion nor is influenced by some of the aforementioned issues, such as *H. Pylori* infection or SIBO. Preliminary data have indeed shown its efficacy in reducing daily OFF time by at least 2 h [28, 109, 110].

**Table 3:** Reduction of daily ‘OFF’ time with continuous levodopa-carbidopa intestinal gel

Study	Number of participants	Average follow-up duration (months)	Reduction of daily ‘OFF’ time (%)
Nilsson et al. [111]	28	37	18
Eggert et al. [112]	13	12	70
Antonini et al. [113]	22	24	46
Antonini et al.[108]	19	14	68
Santos-Garcia et al. [114]	9	170	91
Merola et al. [115]	20	15	68
Fasano et al. [116]	14	25	49
Fernandez et al. [117]	192	21	58
Foltynie et al. [118]	12	12	43
Zibetti et al. [119]	25	36	50
Antonini et al. [120]	98	24	38
Zibetti et al. [121]	59	26	49
Caceres-Redondo et al. [122]	29	24	58
Sensi et al. [123]	28	24	57
Lundqvist et al. [124]	10	12	71
Fernandez et al. [125]	272	12	66
Calandrella et al. [126]	35	32	54
Buongiorno et al. [127]	72	22	56
Slevin et al. [128]	62	13	46
Lopiano et al. [129]	145	14	57
Vallderiola et al. [130]	177	35	66
Merola et al. [131]	20	62	55
Chang et al. [132]	15	12	71
De Fabregues et al. [133]	23	44	82
Antonini et al. [134]	375	24	65
Standaert et al. [135]	38	14	74
Juhasz et al. [136]	34	12	84
Zibetti et al. [137]	32	31	62
Fernandez et al. [138]	86	49	67
Lopiano et al. [139]	145	36	50
Fabbri et al. [140]	44	52	60
Popa et al. [141]	24	12	29
Standaert et al. [142]	195	12	65
Weighted average improvement in OFF time*			59.8

Data from open-label studies assessing the efficacy of continuous levodopa-carbidopa intestinal gel in reducing ‘OFF’ time in the treatment of patients with advanced Parkinson’s disease. Only studies which reported reduction of daily ‘OFF’ time were included

*Weighted for participant number per study

### Determinants of persistent ‘OFF’ periods in CDD therapies

In this review, we have summarised the evidence for the effectiveness of the three most commonly used therapies employed to provide CDD in treating ‘OFF’ periods—transdermal delivery of rotigotine, CSAI and LCIG. The conclusion reached is that even with CDD, significant amounts of ‘OFF’ time remain, appearing unresponsive to dopaminergic therapy. We have examined in detail the potential reasons why each CDD-based non-oral therapy might fail to abolish ‘OFF’ periods, in relation to the technologies underlying drug delivery. In individual patients, these are perfectly viable reasons for the persistence of motor fluctuations and should be addressed to optimise therapy.

However, a number of caveats need to set out before discussing the findings in greater detail:

1. For each therapy, the major clinical parameter used to assess efficacy has relied on ‘OFF’ time, but the definition of ‘OFF’ can be vague and inconsistent and surprisingly, remains poorly defined. The ‘OFF’ time is often based on patients’ Hauser diaries, which might lack accuracy, as for other ‘ON/OFF’ diaries [143]. An alternative option for objective measurement of ‘OFF’ periods is the use of home-monitoring devices, such as Parkinson's KinetiGraph™ (PKG) or KinesiaU (Great Lakes Neurotechnology), whose full potential in the clinical practice is still under investigation [144–146]. Additionally, the quality and the severity of the ‘OFF’ periods, together with the involvement of motor and non-motor components, need more clarity.
2. It is unclear as to whether these therapies improve both predictable and unpredictable ‘OFF’ periods, or whether it is predictable ‘OFF’ periods that respond and unpredictable that persist.
3. The response is highly variable among individual patients and a complete abolition of ‘OFF’ periods can be achieved in some, while others still exhibit marked ‘OFF’ time.
4. It seems unclear whether additional standard oral dopaminergic therapy or a further increase in the dose for CDD, further reduces ‘OFF’ periods, as this may be masked by the severity of dyskinesia.
5. The changes in the temporal pattern of ‘OFF’ periods over the day and over time after introduction of CDD remain to be fully mapped.

Having defined the caveats that complicate the data presented for each of the continuous therapies, there are potentially important conclusions that can be reached by a comparison of their individual profiles. The fact that the continuous therapies involve different drugs with differing modes of action and different routes of administration using different technologies allows for exclusion of the drug delivery parameters set out in Table 4 to be responsible for the persistence of ‘OFF’ time.

**Table 4:** Possible reasons for persistence of ‘OFF’ periods in continuous drug delivery therapies

Reasons for ‘OFF’ periods	Rotigotine	LCIG	CSAI
*Drug- or Device-related*			
Dose/Time	✓	✓	✓
Pump Failure	×	✓	✓
Line Blockage	×	✓	✓
Patch adhesion	✓	×	×
Tube/needle displacement/migration	×	✓	✓
Fibrosis/adhesion	×	✓	✓
*Site-specific*			
Local Peritonitis	×	✓	×
Sub-absorption	×	✓	×
*H. Pylori* infection	×	✓	×
Gastritis	×	✓	×
Duodenitis	×	✓	×
Small intestinal bacterial overgrowth	×	✓	×
Protein-rich meals or fasting	×	✓	×
Constipation	×	✓	×
Skin conditions/skin nodules	✓	×	✓
*Central or Disease-related*			
Brain penetration	?	?	?
Conversion to dopamine	×	✓	×
DA receptors stimulation	✓	✓	✓
Non-DA binding	?	?	?
Presynaptic storage	×	✓	×
Loss of long-duration response	✓	✓	×
Involvement of non-dopaminergic pathways	?	?	?
*Others*			
Infection (e.g., urinary tract infection)	✓	✓	✓
Emotional stressor	✓	✓	✓
Diurnal (circadian) pattern	?	✓	?

*LCGI* levodopa-carbidopa intestinal gel, *CSAI* continuous subcutaneous apomorphine infusion

The major arguments are set out below:

1. Despite the use of differing technologies to provide CDD, the overall finding of persistent ‘OFF’ time is common to all. This suggests that a technology-based explanation is not viable, with the exception of dose/time. A dose-based reason for remaining ‘OFF’ time is feasible, but persistent ‘OFF’ time is seen with differing periods of daily drug delivery for levodopa and apomorphine, and rotigotine is delivered 24 h per day but still fails to remove ‘OFF’ periods.
2. Site-specific reasons for persistent ‘OFF’ periods also appear unlikely based on the differing modes of drug delivery. Only levodopa is administered through the gastro-intestinal tract, and none of the multiple potential reasons underlying remaining ‘OFF’ periods would apply to rotigotine or apomorphine delivery.
3. In contrast to few studies on oral levodopa [147–149], there is no evidence that plasma drug levels change during the day to coincide with remaining ‘OFF’ periods or for other changes in the peripheral pharmacokinetics of levodopa, apomorphine or rotigotine. This leads to the conclusion that the remaining ‘OFF’ periods are uncontrolled because of events occurring within the brain.
4. There is no evidence for altered penetration of drugs into the brain that would coincide with ‘OFF’ periods. Only levodopa shows potential change of active uptake linked to competition from dietary large neutral amino acids [103, 105]. Rotigotine and apomorphine penetrate by simple diffusion [150, 151].
5. Involvement of conversion and storage of dopamine is only relevant to levodopa [152, 153] and does not apply to the dopamine agonists. Similarly, presynaptic storage and the involvement of a long duration response only relate to levodopa [11] and would not explain ‘OFF’ periods resistant to apomorphine or rotigotine.
6. Alterations in dopamine receptor stimulation would provide a common link for the drugs administered by continuous delivery. All are ‘broad-spectrum’ dopamine agonists or levodopa-based and have effects on the major post-synaptic dopamine receptor subtypes—at least in vitro [154]. Changes in dopamine receptor density may occur in PD although the direction of change is disputed. CDD may ‘desensitise’ dopamine receptors, leading to reduced efficacy. Indeed, Quinn and colleagues reported that 24-h intravenous infusion of levodopa leads to a ‘tolerance’ after some days [155]. This has to be balanced against a lack of evidence for development of a similar tolerance after continuous 24-h delivery of rotigotine. However, alterations in receptor sensitivity occur relatively slowly rather than being transient and rapidly reversible, and as such would not explain the resistant short-duration ‘OFF’ periods. An exception to this could be the more rapid changes that are thought to occur in D-1 dopamine receptors linked to alterations in receptor trafficking [156].
7. In LCIG-treated patients, worsening of parkinsonian symptoms and ‘OFF’ periods occur mainly in the afternoon/evening despite no change in levodopa plasma level [107]. A similar change in the duration of response also occurs in response to apomorphine bolus injections [157], although it is unknown whether this is also seen with subcutaneous infusions of apomorphine. A role for physiological diurnal patterns might explain this phenomenon since motor function in patients with PD fluctuates throughout the day, often being worse in the afternoon [158–161] even in de novo PD [162], suggesting that this motor fluctuation is independent of dopaminergic medication. But this does not reflect the clinical reality of continuing unpredictable ‘OFF’ periods seen when using CDD.

### Possible explanations behind the persistence of ‘OFF’ periods during CDD therapies

All of the above increasingly seems to lead to the conclusion that the causes of persistent ‘OFF’ periods are not the drugs or the delivery technology, but rather the disease itself. It appears that despite the best efforts being made to deliver drugs in compliance with the concepts of CDD and CDS [163], dopaminergic therapy is not able to control the persistent ‘OFF’ periods that remain. An obvious explanation for this, is that the persistent ‘OFF’ periods are non-dopaminergic in origin. At first glance, this would seem to challenge the accepted view of ‘wearing OFF’ as a common manifestation of motor fluctuations being due to the loss of presynaptic dopaminergic terminal storage, related post-synaptic dopamine receptor changes and responsiveness to improved dopaminergic drug delivery. However, another view would be that there are predictable ‘OFF’ periods, such as ‘wearing OFF’ which are dopaminergic in nature and respond to continuous delivery therapies, but that in addition, there are unpredictable ‘OFF’ periods that are non-dopaminergic in origin and do not respond to CDD. The non-dopaminergic basis for some ‘OFF’ periods would not be out of line with similar views on the underlying cause of motor complications in PD, notably dyskinesia being due to changes in basal ganglia circuitry beyond the dopaminergic inputs [164, 165].

If persistent ‘OFF’ periods are non-dopaminergic in origin, then a non-dopaminergic approach to treating them should be explored, in the same way that non-dopaminergic treatments have been evaluated for dyskinesia, with a role for glutamatergic, noradrenergic, and serotonergic pathways among others [165, 166]. For example, amantadine, zonisamide, istradefylline and safinamide, which have a mixture of pharmacological actions that are non-dopaminergic in nature, all reduce ‘OFF’ time when used as an adjunct to dopaminergic therapies [68–70, 167–175]. This illustrates that the occurrence of motor fluctuations and ‘OFF’ periods is largely beyond than can be explained simply by inadequate stimulation of dopaminergic function.

The conclusion of the evaluation of the use of CDD in early and late PD in relation to ‘OFF’ periods that appear resistant to further alterations in dopaminergic therapy, is that to explain their presence we need to look beyond dopamine and dopaminergic therapies. As a final caveat, it should be noted that the proposed explanation for persistent ‘OFF’ periods has at least one substantial flaw, that is related to the fact that they can be explained with a single mechanism across everyone affected. An alternative view might be that they have multiple causes, differentially applicable across the biological universe of PD. Future studies should also investigate whether introducing CDD at an early point in PD—before motor fluctuations and motor complications occur—would prevent their development and as a result, the drug-resistant ‘OFF’ periods would not become the problem that they currently pose.

### A speculative view of future investigation of ‘OFF’ periods

While it is always good to challenge existing concepts of the complexity of PD, it is also necessary to provide directions for future investigation. This review has questioned the current view that ‘OFF’ periods are purely dopaminergic in nature, but does not rule out some manifestations of ‘OFF’ such as ‘wearing OFF’ being due to altered pharmacokinetic or pharmacodynamic responses to levodopa—although whether this would apply to dopamine agonists as well is unknown. But what it does raise is the question of the pathophysiology of ‘OFF’ periods in general, which is an area that needs more basic research. While much emphasis has been placed on understanding dyskinesia at the cellular and molecular level [171, 172], the same degree of investigation has not been given to ‘OFF’ periods even though they are at least an equally common clinical problem and an area of unmet pharmacological need. It is plausible to argue that abnormal signalling within the striato-thalamo-cortical loop contributes to ‘OFF’ periods in the same way as postulated for dyskinesia [171], but this has not been examined. A significant problem is that there are not sufficiently adequate experimental models of ‘OFF’ periods in the manner available for dyskinesia [173, 174]. This may tell us that using ‘simple’ dopaminergic denervation to model PD is not sufficient to induce those changes that lead to ‘OFF’ periods. But there has been so far, relatively little interest in exploring more widespread pathological changes in animal models as a way of understanding ‘OFF’ periods. It is quite possible that an imbalance between monoaminergic transmitters (dopamine, noradrenaline and serotonin) could be at the heart of neural network disruption leading to ‘OFF’ periods as all are known to be involved in the control of motor function and probably dyskinesia [164, 165, 175]. What is clear from the use of a range of non-dopaminergic drugs, including some that alter serotoninergic, noradrenergic, glutamatergic, cholinergic and adenosine transmission (as detailed earlier), is that while these can decrease ‘OFF’ time, no single pharmacological manipulation has yet been shown to eliminate ‘OFF’ periods. Perhaps we need to revert to the use of ‘dirty’ drugs that would influence multiple neurotransmitters affected by PD if ‘OFF’ periods are to be controlled.

A final but highly relevant question that requires investigation is the meaning of ‘OFF’ periods, whether a redefinition is in order. First, ‘OFF’ periods are a catch-all term that is routinely employed to cover unexplained immobility. We have highlighted the difference between ‘predictable’ and ‘unpredictable’ ‘OFF’ but there seems to be virtually no detailed clinical investigation of the characteristics and temporal components of ‘OFF’ periods in recent times. For example, we have highlighted the effects of CDD on ‘OFF’ periods, but despite the increasingly common use of advanced therapies, there seems no study investigating the changes of pattern, intensity, and temporal occurrence of ‘OFF’ periods in individual patients before versus during CDD. This investigation is relatively easy to undertake and would produce meaningful outcomes to advance our understanding of those ‘OFF’ periods unresponsive to dopaminergic medication; however, the requirement of a levodopa pharmacokinetic profile creates logistical and funding difficulties. Second and perhaps most provocatively, is what we regard as ‘OFF’: is it a pure manifestation of a failure of voluntary movement due to altered basal ganglia function or is it something else? Is it some other dysregulation of movement, a loss of cortical command, a manifestation of ‘freezing’? It could be that the ‘OFF’ periods relate to the occurrence of specific non-motor symptoms of PD—apathy, lethargy, depression, impaired cognition—and we may miss important clues to the cause or causes of one of the most troublesome deficits in current treatment of PD. Lastly, it is important to acknowledge that ‘OFF’ and ‘ON’ are arbitrarily dichotomized aspects of the experience of PD patients who rarely endorse switch-like changes between these theoretical states. Instead, they experience “shades” of ‘OFF’ and ‘ON’ along a continuum whose artificial borders were created as endpoints in clinical trials. Future integration of technologies to measure motor and non-motor fluctuations in the patients’ own environment may well replace the rigid construct of ‘OFF’ and ‘ON’ and prompt the revisitation of the issues noted here from a patient-centric perspective.

## Conclusions

‘OFF’ periods during CDD remain one of the biggest challenges in the care and treatment of patients with PD. More studies are needed to better characterize and understand this phenomenon, whose pathogenesis seems complex and beyond the simple dopaminergic dysfunction hypothesis [176].

## Data Availability

Not applicable.

## Abbreviations

PD: Parkinson’s disease
CDD: Continuous drug delivery
CDS: Continuous dopaminergic stimulation
CSAI: Continuous subcutaneous apomorphine infusion
LCIG: Levodopa-carbidopa intestinal gel
SIBO: Small intestinal bacteria overgrowth
